# Carvacrol alleviates the proconvulsive effects of lipopolysaccharide (LPS) and reduces the gene expression of proinflammatory cytokines *interleukin-1 *and *tumor necrosis factor-α*

**DOI:** 10.22038/ajp.2025.25518

**Published:** 2025

**Authors:** Hesamodin Bagheripoor, Mahdieh Mondanizadeh, Mehdi Sadegh

**Affiliations:** 1 *Faculty of Medicine, Arak University of Medical Sciences, Arak, Iran*; 2 *epartment of Biotechnology and Molecular Medicine, Faculty of Medicine, Arak University of Medical Sciences, Arak, Iran*; 3 *Department of Physiology, Faculty of Medicine, Arak University of Medical Sciences, Arak, Iran*

**Keywords:** Inflammation, Interleukin 6 (IL-6), Interleukin 4 (IL-4), Seizure

## Abstract

**Objective:**

Carvacrol has anti-inflammatory effects. According to the links between inflammatory processes and seizures, this study was designed to investigate the potential effect of carvacrol in reducing seizure severity and involvement of hippocampal pro- and anti-inflammatory cytokines.

**Materials and Methods:**

This research was conducted on 42 adult male Wistar rats. Animals were randomly divided into seven groups (n=6). Seizures were induced by PTZ (Pentylenetetrazol) injection (80 mg/kg). LPS (lippolysaccharide) was injected (400 μg/kg) 4 hr before PTZ. Carvacrol (100 mg/kg) was injected immediately after LPS. All injections were intraperitoneal (i.p.). Experimental groups were as follows: 1. Control (Cnt) 2. Carvacrol (Cav); 3. LPS; 4. PTZ, 5. PTZ+LPS; 6. PTZ+Cav; 7. PTZ+LPS+Cav. Seizures were observed for 30 min after the PTZ injection and the occurrence of behavioral stages of seizures was recorded. Following the behavioral study, hippocampal samples were collected for gene expression evaluation using the Real Time-PCR technique to assess IL (interleukin)-1, IL-6, IL-4 and TNF (tumor necrosis factor)-α gene expression.

**Results:**

The current study showed that receiving LPS exacerbated seizures in the studied groups. Carvacrol reduced the severity of seizures in the LPS-receiving groups. In the gene expression study, receiving LPS increased the expression of cytokines TNF-α and IL-1 in the hippocampal tissue. Carvacrol significantly decreased gene expression of TNF-α and IL-1.No significant changes were detected for IL-6, IL-4 gene expression.

**Conclusion:**

There could be a relationship between carvacrol ability to modulate the proconvulsive effects of LPS and its ability to decrease the gene expression of inflammatory cytokines.

## Introduction

Epilepsy is a prevalent neurological disease described byrecurrent seizures. It is estimated that 50 millionpeople worldwide have epilepsy (Fiest et al. 2017). It is reported that 30-35% of epilepsy cases do not respond adequately to current anti-epileptic drugs (Loscher et al. 2020). By examining samples obtained from the brains of epilepsy patients, it appears that changes in hippocampal neurons cause the loss of balance between the excitation and inhibition in the limbic system leading to subsequent spontaneous seizures (Hoogland et al. 2000). Traumatic or non-traumatic brain injuries such as seizures trigger biological chains that release cytokines in the space around neurons (Vezzani et al. 2013). 

Previous reports suggest that inflammation and immune-related responses such as interleukins in brain tissue could increase the risk of convulsions, making these factors potential biomarkers of seizure susceptibility (Gorter et al. 2006). Cytokines are inflammation-related proteins produced and released in the early stages by glial cells in the neuronal environment, and they can affect neuronal excitability and other glial cell functions (Shimada et al. 2014). 

In clinical studies, seizures caused by fever have shown an increase in cytokines interleukin-1 (IL-1), interleukin-6 (IL-6), and tumor necrosis factor-α (TNF-α) in the cerebrospinal fluid (Dinarello 2004). TNF-α is an inflammatory cytokine which is released by activated microglia and causes an increase in glutamate neurotransmission and neuronal excitation (Stellwagen and Malenka 2006). IL-1 is expressed by microglia and causes a reduction in glutamate uptake. The increase in this cytokine after seizure causes dysfunction of neurons and decreases their regeneration capacity (Alyu and Dikmen 2017). IL-6 is usually detectable in small amounts in the nervous system, but the stimulation of astrocytes and microglia cells could increase its production. Studies on the effect of this cytokine on convulsions are conflicting (Gruol 2015). IL-4 is an anti-inflammatory cytokine with a proven effect in reducing the activity of various parts of the immune system by inhibiting the production of pro-inflammatory cytokines (Chen et al. 2020).

Carvacrol (C10H14O) is abundantly found in the *Lamiaceae* family, including thymus, oregano, and savory, which are a. Studies have shown that phenolic plant compounds like carvacrol have anti-inflammatory properties and can reduce pro-inflammatory cytokines (Sadegh and Sakhaie 2018). 

In this study, our goal was to investigate if carvacrol is able to alleviate proconvulsive effects of lipopolysaccharide (LPS). To understand the cellular pathways and genes involved in these effects, we investigated the effects of carvacrol on gene expression of proinflammatory cytokines IL-1, IL-4, IL-6 and TNF-α.

## Materials and Methods

### Animals

Forty-two adult male Wistar rats (180–200 g) were used for this research. All rats were placed in standard cages and standard conditions (temperature of 25°C ، 12 hr light/12 hr darkness). Food and water were freely accessible. All provision from “Guide for the Care and Use of Laboratory Animals” (8th edition; National Academies Press; 2011) was considered. All efforts were made to reduce the number of animalsand minimize their suffering.

### Chemicals and seizure induction

Carvacrol, Pentylenetetrazol and LPS (obtained from Gram-negative *Escherichia coli* serotype O111:B4) all were from Sigma Aldrich (USA). Carvacrol (100 mg/kg) was dissolved in the DMSO (dimethyl sulfoxide) 1% and was injected intraperitoneally (i.p.) immediately after LPS. According to our previous works DMSO 1% had no effect on seizure parameters. LPS (400 μg/kg) and PTZ (80 mg/kg) were dissolved insaline (0.9%) and injected i.p. (Akarsu et al. 2006; Zareie et al. 2018). 

Behavioral seizure scales were monitored for 30 min following the injection of PTZ. For this, each rat was placed into a Plexiglass made box (40*40*40 cm) immediately after receiving the injections. 

Seizure stages were scaled as follows: stage 0, normal behavior; stage 1, sniffing and/or freezing; stage 2, head nodding, clonus in head muscles and forelimbs; stage 3, continuous myoclonic jerk, tail rigidity; stage 4, bilateral clonus in forelimbs and jumping; stage 5, tonic-clonic convulsions, rearing and falling. By observing and recording time using a digital timer, the duration of reaching each stage, the duration of stages 3 to 5, the number of occurrences of each stage and the death rate in animals weremeasured and recorded for additional analysis.

### Experimental groups

The studied rats were divided into seven groups of 6 as follows: 

1. Control (Cnt) 2. Carvacrol (Cav), receive only carvacrol; 3. LPS, receive only LPS; 4. PTZ, receive only PTZ, 5. PTZ+LPS, PTZ was injected four hours after receiving LPS; 6. PTZ+Cav, PTZ was injected four hours after carvacrol; 7. PTZ+LPS+Cav, PTZ was injected four hours after carvacrol and LPS.

Seizure stages were measured in those PTZ-treated groups. After conducting behavioral seizure tests, animals were sampled for real-time PCR test. First, animal head wasquickly removed under chloroform anesthesia using a guillotine. To induce anesthesia, animal were placed in a see-through box, containing chloroform. Then, the skull was opened along the midline and the brain was extracted. On an ice-cooled cutting board, the left and right hippocampus was dissected from hemispheres,snap frozen in liquid nitrogen and stored at −70^◦^C for extraction of DNA and RNA. 

### RNA isolation, DNA digestion, and reverse transcription

RNA was extracted from the hippocampus (both left and right hippocampus were mixed) using RNX-plus kit of Cinaclone Company and the work steps were carried out according to the manufacturer's instructions. Spectrophotometric optical density measurement (260 and 280 nm) was used to determine RNA total concentration. For each sample tested, the ratio the spectrophotometric readings ratio at 260 nm and 280 nm (OD260/OD280) was used as estimation for nucleic acid purity. The ratios for all samples were between 1.7 and 2.0. 

Oligo(dT) primers were used for reverse transcriptase reactions. Each reaction tube contained 50 ng/µg of total RNA, 11 μl of cDNA Synthesis MIX ،0.5μmol/L of random hexamers and diethyl dicarbonate (DEPC(-treated water to volume. A semi-quantitative reverse transcriptase polymerase chain reaction (RT-PCR) was performed to determine the levels of *TNF-α, IL-4, IL-6, IL-1* mRNA expressions. A DNA Thermal Cycler 480 (Perkin Elmer, Branchburg, NJ, U.S.A.) was used for reverse transcriptase reactions. Reactions were completed at 42°C for 20 min and 99°C for 5 min. The final cDNA was transferred to −20°C.

The sequence of TNF-α, IL-4, IL-6, IL-1 genes was extracted from the NCBI database. The primers used to measure the expression of the mentioned genes were designed by Allele ID software and after performing BLAST in the NCBI database was synthesized by Cinaclone.

### Statistical analysis

Data were analyzed with GraphPad Prism 5.0. One-way ANOVA and Tukey post-test were used to check the difference amongthe groups. Chi-square test was usedfor mortality rateanalysisand a p<0.05 was considered significant. All data are presented as a mean±SD.

## Results

### Seizure data

Latency to each seizure stage was analyzedamong thegroups by one-way ANOVA. As demonstrated in Table 1 latencies to stages 3, 4 and 5 in LPS+PTZ weresignificantly lower than PTZ (stage 3 p<0.01; stages 4 and 5 p<0.05), which means that LPS+PTZ group wasmore susceptible for seizure compared to the PTZ group. However, carvacrol injection in the LPS+Cav+PTZ group significantly reduced this effect of LPS and significantly increased latencies to stages 3, 4 and 5 compared with LPS+PTZ group (in both stages 3 and 5 p<0.01; in stage 4 p<0.05). In addition, carvacrol injection in the PTZ group provided a significant increase in stage 3 latency (p<0.05) but had no significant effect on other stages. So, it seems that carvacrol was more effective on LPS proconvulsive effect than on PTZ-induced seizures. 

Moreover, chi-square analysis for mortality rate following the tonic-clonic convulsion on stage 5, showed a higher death rate in the LPS+PTZ group (3 rats died out of 6 rats) than the PTZ group (1 rats died out of 6 rats) (p<0.01). In other groups, no death happened after the stage 5 (Table 1). 

As no significant effect wasappeared on latencies to stages 1 and 2, we did not report these data in Table 1. 

### Gene expression data

**Figure 1:**
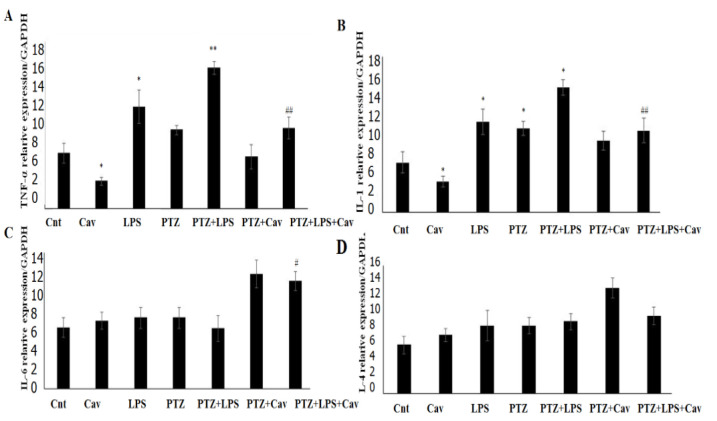
*Carvacrol eliminated LPS increasing effect on *TNF-α* and *IL-1* gene expression. *TNF-α* (A) and *IL-1 (B) were significantly increased in the LPS and PTZ+LPS groups compare to the control (Cnt), while significantly decreased in PTZ+LPS+Cav group compare to PTZ+LPS. Data is presented as Mean±SD; n= 6/groups; *p<0.05 and **p<0.01 compared with Cnt, ##p<0.01 compared with PTZ+LPS.

As demonstrated in Figure 1, hippocampus gene expression of pro-inflammatory cytokines *TNF-α* (A) and *IL-1* (B) wassignificantly increased in the LPS and PTZ+LPS groups compared to the control (Cnt) (for *TNF-α* p<0.05 for the LPS group and p<0.01 for the PTZ+LPS group compared to Cnt; for *IL-1* p<0.05 for both LPS and LPS+PTZ groups compared to Cnt), while both *TNF-α* and *IL-1* gene expression significantly decreased in the carvacrol (Cav) group compared to the control (p<0.05 for both genes). In addition, gene expression of these pro-inflammatory cytokines in the PTZ+LPS+Cav group significantly decreased compared to the PTZ+LPS group (p<0.01 for both genes), which means that carvacrol eliminates increasing effect of LPS on gene expression of these two proinflammatory cytokines.

Moreover, anti-inflammatory cytokines *IL-4* and *IL-6* gene expression (Figure 1-C, D) revealed no significant changes in compare with LPS or carvacrol in the Cav, LPS and PTZ+LPS groups compared to the control, although *IL-6* expression in the PTZ+LPS+Cav group wassignificantly increased compared to the PTZ+LPS group (p<0.05). 

**Table1:** Latencies to seizure stages 3-5.

**Dead out of 6 rats**	**S5L(s)**	**S4L(s)**	**S3L(s)**	**Groups**
1	542±82	404±80	219±51	PTZ
3**	343±88 *	256±64 *	134±30 **	LPS+PTZ
0	664±199	500±144	277±56 *	Cav+PTZ
0	550±107 ##	419±133 #	208±34 ##	LPS+Cav+PTZ

LPS significantly decreased latency (s) to seizure stages 3-5, while carvacrol prevented this effect of LPS. Number of death after stage 5 is demonstrated. Data as Mean±SD; *p<0.05 and**p<0.01 compared to PTZ; #p<0.05 and##p<0.01 compared with LPS+PTZ.S3L: latency to stage 3, S4L: latency to stage 4, S5L: latency to stage 5, PTZ: Pentylenetetrazol, LPS: Lippolysaccharide, Cav: Carvacrol.

## Discussion

In our study, results demonstrated carvacrol efficiency to reduce LPS proconvulsive effects in the tonic-clonic seizure induction model induced by PTZ. In conditions without LPS, carvacrol also increased the latencies to stages 2–3 of seizure which are similar with focal seizures. Gene expression analysis revealed that the anticonvulsant effect of carvacrol could be accompanied with a decrease in cytokines gene expression in the nervous system. Previous studies have shown that LPS injection facilitates PTZ-induced seizures (Sayyah et al. 2003). The proconvulsant effect of LPS begins approximately 30 min after administration and can last up to 12 hr. Seizure susceptibility increases 4 hr after LPS injection with a dose of 400 μg/kg in rats (Akarsu et al. 2006). Based on these findings, we also administered LPS 4 hr before PTZ at a dose of 400 μg/kg. Consistent with earlier reports, our results indicated that LPS exacerbates acute seizure indexes and acts as a proconvulsant agent. Xiang et al. demonstrated that LPS can activate the toll like receptor-4 (TLR4) signaling pathway, leading to excessive release of inflammatory cytokines (Xiang et al. 2016). Although systemic LPS cannot directly reach the brain, Ho et al. reported that proinflammatory cytokines induced by LPS can cross the blood brain barrierand increase susceptibility to seizures (Ho et al. 2015). In our study, a significant increase in cytokines IL-1, IL-4, IL-6, and TNF-α was observed after LPS administration in the hippocampus.

In seizure models induced byPTZ, an increase in the levels of IL-1β, IL-6, and TNF-α cytokines in the hippocampus of rats has been reported (Singh et al. 2019). Our results also indicated an increase in the expression of cytokines IL-1, IL-4, IL-6, and TNF-α in the group receiving PTZ compared to the control group. Given the chronic nature of epilepsy, there is sufficient time for inflammatory reactions to activate. Neuroinflammation, by affecting nerve excitability, can lead to the facilitation of convulsions (Dey et al. 2016; Vezzani et al. 2011). Fiala et al. reported that serum levels of IL-1, TNF-α, and IL-6 were elevated in patients with temporal lobe epilepsy and refractory epilepsy (Fiala et al. 2013). Similarly, levels of cytokines IL-1α, TNF-α, and IL-6 increased in neurons and glial cells of the nervous system before the onset of seizures (Frantz et al. 2017).

Ho et al. suggested that using anti-inflammatories could be a therapeutic approachto reduce seizures provoked by environmental inflammatory factors (Ho et al. 2015). Carvacrol is a herbal compound with confirmed anti-inflammatory effects in various studies. It has been reported that carvacrol's neuroprotection is primarily related to its antioxidant and anti-inflammatory effects (Azizi et al. 2022). Mahmoodi et al. investigated the effect of carvacrol on white brain tissue in experimental autoimmune encephalitis (EAE) mice and observed that carvacrol modulates the expression of pro-inflammatory and anti-inflammatory cytokines in the CNS (Mahmoodi et al. 2019). Carvacrol has been shown to reduce the production of prostaglandins by suppressing cyclo-oxygenae-2(Sadegh and Sakhaie 2018). Consistent with our data, previous reports have shown the protective function of carvacrol against the inflammatory and brain damaging effects of LPS. In these studies, carvacrol increased antioxidant factors like malondialdehyde anti-inflammatory factors like TNF-α andIL-6 (Hakimi et al. 2020; Salmani et al. 2022). Additionally, systemic inflammation and oxidative effects of paraquat were reduced by carvacrol administration in a dose similar to our study, with involvement of MDA, IL-6, and Interferon-γ (Amin et al. 2020a; Amin et al. 2020b).

Furthermore, the anticonvulsant effects of carvacrol have been investigated in different animal models of seizure. In the study by Khalil et al., carvacrol at a dose of 70 mg/kg suppressed seizures in an electroconvulsive model. They concluded that carvacrol prevented the occurrence of status convulsions due to its antagonistic effect on the transient receptor potential melastatin7 (TRPM7) channels (Khalil et al. 2017). Another study also demonstrated the anticonvulsant effect of carvacrol in a maximal electroshock model (Mishra and Baker 2014). In our PTZ -induced convulsion model, the proconvulsive effects of LPS disappeared after carvacrol administration. In biochemical evaluations, carvacrol decreased the cytokines IL-1 and TNF-α and increased the expression of IL-6 and IL-4 in the LPS group compared to the control group. Carvacrol also produced a similar effect in the PTZ+carvacrol group compared to the PTZ group. Interestingly, results from a clinical trial study showed that daily oral administration of carvacrol (1-2 mg/kg) for one month had no negative effect on physiological indexes of the respiratory and blood system (Ghorani et al. 2019).

The exacerbation of inflammation in the nervous system after LPS and PTZ administration can explain the increase in seizures in the LPS+PTZ group compared to the PTZ group. Although IL-1 and TNF-α are recognized as inflammatory cytokines, there is no definite consensus on the inflammatory or anti-inflammatory effect of IL-4 and 6. In our study, it appears that cytokines IL-1 and TNF-α act as inflammatory cytokines, while IL-4 and IL-6 suppressed inflammation and acted as anti-inflammatory cytokines. Therefore, it can be concluded that carvacrol reduces seizures by increasing anti-inflammatory cytokines IL-4 and IL-6 and suppressing inflammatory cytokines TNF-α and IL-1.

 It appears that LPS exacerbates seizures by increasing the expression of inflammatory cytokines in the central nervous system. Our study demonstrated that carvacrol decreases the expression of inflammatory factors IL-1 and TNF-α and increases the expression of cytokines IL-4 and IL-6 in the central nervous system. This is one potential pathway through which carvacrol suppresses the pro-convulsing effect of LPS. Therefore, carvacrol could be used as an anti-inflammatory adjunct to existing anti-epileptic treatments toimprove seizure control. However, further research and experiments are necessary in this field.
